# Frequency Dependency of the Delta-E Effect and the Sensitivity of Delta-E Effect Magnetic Field Sensors

**DOI:** 10.3390/s19214769

**Published:** 2019-11-02

**Authors:** Benjamin Spetzler, Elizaveta V. Golubeva, Cai Müller, Jeffrey McCord, Franz Faupel

**Affiliations:** Kiel University, 24118 Kiel, Germany; besp@tf.uni-kiel.de (B.S.); elgo@tf.uni-kiel.de (E.V.G.); camu@tf.uni-kiel.de (C.M.); jmc@tf.uni-kiel.de (J.M.)

**Keywords:** delta-E effect, magnetoelasticity, resonators, magnetic field sensing, dynamic susceptibility, surface acoustic wave (SAW)

## Abstract

In recent years the delta-E effect has been used for detecting low frequency and low amplitude magnetic fields. Delta-E effect sensors utilize a forced mechanical resonator that is detuned by the delta-E effect upon application of a magnetic field. Typical frequencies of operation are from several kHz to the upper MHz regime. Different models have been used to describe the delta-E effect in those devices, but the frequency dependency has mainly been neglected. With this work we present a simple description of the delta-E effect as a function of the differential magnetic susceptibility χ of the magnetic material. We derive an analytical expression for χ that permits describing the frequency dependency of the delta-E effect of the Young’s modulus and the magnetic sensitivity. Calculations are compared with measurements on soft-magnetic ${\left({\mathrm{Fe}}_{90}{\mathrm{Co}}_{10}\right)}_{78}{\mathrm{Si}}_{12}B_{10}$ thin films. We show that the frequency of operation can have a strong influence on the delta-E effect and the magnetic sensitivity of delta-E effect sensors. Overall, the delta-E effect reduces with increasing frequency and results in a stiffening of the Young’s modulus above the ferromagnetic resonance frequency. The details depend on the Gilbert damping. Whereas for large Gilbert damping the sensitivity continuously decreases with frequency, typical damping values result in an amplification close to the ferromagnetic resonance frequency.

## 1. Introduction

The change of the effective elastic properties with magnetization is referred to as the delta-E effect. It results from inverse magnetostriction that adds an additional strain to the purely elastic Hookean strain [1,2,3]. The delta-E effect has been used to build various types of magnetic field sensors for the detection of low frequency and low amplitude magnetic fields. The first integrable devices [4] were achieved using magnetoelectric MEMS (Micro-Electro-Mechanical Systems) cantilevers [5,6,7] and nano-plate resonators [8,9,10] with thin soft-magnetic layers from 100 nm–2 µm. These structures are excited electrically via a piezoelectric layer to oscillate at their respective resonance frequencies. Typical operation frequencies are from several kHz up to several hundred MHz [11]. Upon application of a magnetic field, the Young’s modulus of the magnetostrictive layer changes, which detunes the resonance frequency. Similar kinds of delta-E effect sensors are based on surface acoustic wave (SAW) devices [12,13,14,15,16,17]. Rayleigh or Love waves are excited with interdigital electrodes at MHz frequencies up to the low GHz regime. The surface waves propagate through a magnetic thin film which is deposited on top of the delay line. Upon application of a magnetic field, the delta-E effect of the magnetic material results in a delay of the surface wave which can be detected as a phase change at the output electrodes. Both kinds of delta-E effect sensors require an external or internal magnetic bias field to be operated at their optimum sensitivities. Besides sensor applications, the delta-E effect has been used for tunable SAW devices operating up to the GHz range [18,19,20,21,22,23,24].

Early experiments and models on the frequency dependency of the delta-E effect focused mainly on polycrystalline nickel rods. In these structures, a strong decrease (from 20% down to 3%) of the delta-E effect was found by an increase of the frequency from a few kHz up to 10 MHz [25,26]. The good agreement with calculations showed that this phenomenon can be assigned to eddy current damping: at low frequencies, micro eddy currents increasingly impede domain wall motion until also the moment rotation is being damped [27]. These calculations are made for bulk structures and are limited to the demagnetized state. The models are based on a quasi-static approach and are only valid for frequencies well below the ferromagnetic resonance frequency.

Despite the first theoretical attempts and the interest in high frequency devices, the existing delta-E effect models [2,28,29,30,31,32,33,34,35] are mainly quasi-static single-spin approaches. Dynamic magnetoelastic models treat the wave velocity [36,37,38] of Rayleigh waves and the electrically driven ferromagnetic resonance [39,40]. Neither calculations for the dynamic delta-E effect nor for the sensitivity for delta-E effect sensors are available.

Here we present a simple but general approach to include the frequency dependency in calculations of the delta-E effect of the Young’s modulus. It is applied to the example of the high frequency regime using a linearization of the magnetization dynamics. Calculations of the dynamic susceptibility are compared with measurements on soft-magnetic material, which has been used for magnetic field sensing before. The resulting parameters are used to calculate the Young’s modulus E(H,f) as a function of magnetic field H and operation frequency f. From E(H,f) the magnetic part $\partial_{E,H}≔\partial E/\partial H$ of the sensitivity $S\propto \partial_{E,H}$ is calculated for resonators dominated by the Young’s modulus. Finally, the results are compared with measurements from the literature.

## 2. The Delta-E Effect

In the following section, a simple equation for the delta-E effect as a function of the differential susceptibility and the magnetoelastic properties is derived and illustrated. Next, an expression for the dynamic differential susceptibility is presented that permits us to describe the frequency and magnetic field dependency of the Young’s modulus.

### 2.1. The ΔE-Effect

The change of Young’s modulus E with the magnetic field results from a change of the magnetoelastic response upon magnetization. Applying a stress σ to the magnetic material results in a purely mechanical strain $e=E_{m}^{-1}\sigma$, inversely proportional to Young’s modulus $E_{m}$ at fixed magnetization. The mechanical strain e is superposed by a stress induced magnetostrictive strain λ that depends on the magnetization M. The total Young’s modulus can be described by [3]

(1) $$\frac{1}{E}=\frac{\partial \left(e+\lambda \right)}{\partial \sigma }≔\frac{1}{E_{m}}+\frac{1}{\Delta E}\;\mathrm{with}\;\frac{1}{\Delta E}≔\frac{\partial \lambda }{\partial \sigma }=\frac{\partial \lambda }{\partial H}\frac{\partial H}{\partial M}\frac{\partial M}{\partial \sigma }.$$

This expression is rearranged to describe the delta-E effect as a function of easy to measure magnetic properties. Using the relation $\partial M/\partial \sigma =\left(1/\mu_{0}\right)\partial \lambda /\partial H$ [41] to replace ∂M/∂σ, the equation becomes

(2) $$\frac{1}{\Delta E}=\frac{{\left(\partial \lambda /\partial H\right)}^{2}}{\mu_{0}\partial M/\partial H}≔\frac{\chi_{\mathrm{me}}^{2}}{\mu_{0}\chi },$$

which is consistent with [3]. Consequently, the change of Young’s modulus is inversely proportional to the square of the differential magnetoelastic susceptibility $\chi_{\mathrm{me}}≔\partial \lambda /\partial H$, with the differential magnetic susceptibility χ≔∂M/∂H as a proportionality factor.

**Figure 1:**
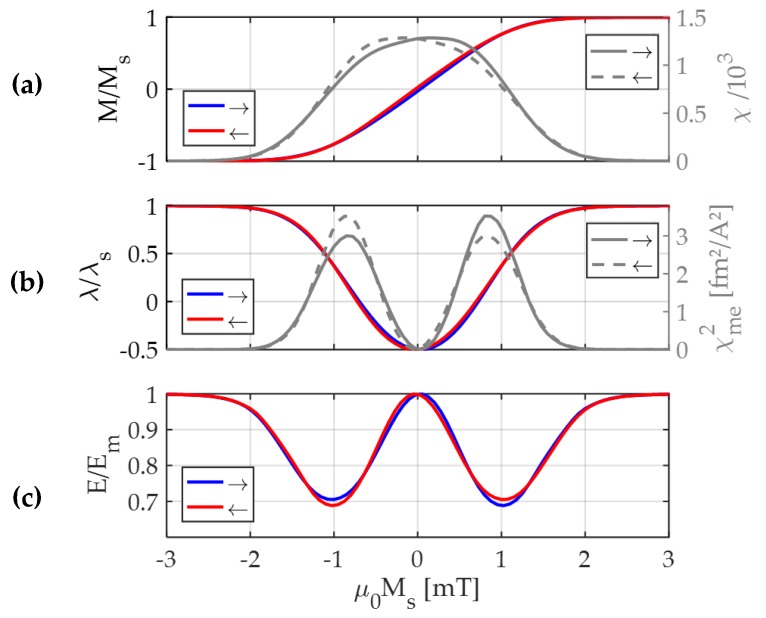
Illustration of the proportionality of the delta-E effect to the squared magnetoelastic susceptibility $\chi_{\mathrm{me}}^{2}≔{\left(\partial \lambda /\partial H\right)}^{2}$ in Equation (2) using a mean-field model based on a single-spin ensemble (Appendix A). (**a**) Modelled magnetic hysteresis curve normalized to the saturation magnetization $M_{s}$ and the differential magnetic susceptibility χ; (**b**) modelled magnetostriction curve, normalized to the magnetostriction $\lambda_{s}$ at magnetic saturation and its squared derivative $\chi_{\mathrm{me}}^{2}$; (**c**) resulting Young’s modulus E as function of the applied magnetic field H, normalized to its value $E_{m}$ at fixed magnetization. The mean-field model and the parameters used for the simulation are given in the Appendix A.

The relation between M(H), λ(H) and E(H) is illustrated in Figure 1 in the example of a soft magnetic amorphous FeCoSiB thin film with uniaxial magnetic anisotropy, typical for cantilever delta-E effect sensors [7]. A mean-field model based on a single-spin ensemble is used to describe λ(H) and M(H), required for Equation (2). Details of the model and the material parameters are given in the Appendix A. A slight hysteresis occurs in M(H), λ(H) and E(H) due to a dispersion of the magnetic easy axis introduced in the model. Note that the minimum of E(H) is at slightly larger fields than the maximum of $\chi_{\mathrm{me}}^{2}$. This occurs because $\chi_{\mathrm{me}}^{2}$ is divided in Equation (2) by $\mu_{0}\chi$, which is maximum around H = 0. The calculation in Figure 1 is consistent the measurements [42] and the trend reported in the literature [29,43].

### 2.2. Frequency Dependency of the Young’s Modulus

To describe the frequency dependency of the delta-E effect, Equation (2) cannot be used directly, because both $\chi_{\mathrm{me}}$ and χ are functions of the magnetization. Instead, the magnetic field and magnetization dependency of $\chi_{\mathrm{me}}$ are separated to describe E as function of χ only. For that, we first use the common quadratic approximation of λ [44] to form the derivative of the magnetostrictive strain to the magnetization

(3) $$\lambda =\frac{3}{2}\lambda_{s}\left(m_{0}^{2}-\frac{1}{3}\right)\to \frac{\partial \lambda }{\partial M}=\frac{1}{M_{s}}\frac{\partial \lambda }{\partial m_{0}}=\frac{3\lambda_{s}m_{0}}{M_{s}}\;.$$

In this equation, $m_{0}$ is the projection of the normalized quasi-static magnetization vector ${\bar{m}}_{0}$ on the axis of the applied static bias field $\bar{H}$. The expression $\chi_{\mathrm{me}}≔\partial \lambda /\partial H=\partial \lambda /\partial M\cdot \;\partial M/\partial H$ is substituted into Equation (2) with ∂λ/∂M from Equation (3), which results in

(4) $$\frac{1}{\Delta E}=\frac{\chi }{\mu_{0}}{\left(\frac{\partial \lambda }{\partial M}\right)}^{2}=\frac{9\lambda_{s}^{2}m_{0}^{2}}{\mu_{0}M_{s}^{2}}\chi \;.$$

Then the Young’s modulus E(H,f) finally is

(5) $$E\left(H,f\right)={\left(\frac{1}{E_{m}}+\frac{1}{\Delta E\left(H,f\right)}\right)}^{-1}\;\mathrm{with}\;\frac{1}{\Delta E}=\frac{9\lambda_{s}^{2}m_{0}^{2}}{\mu_{0}M_{s}^{2}}\chi \;.$$

The magnetostrictive strain λ(M) results from the strain response of the mechanical structure to the magnetization induced magnetoelastic stress. Measurements and simulations on FePt nanoparticles indicate that this local structural response occurs on the timescale of a few pico-seconds [45]. Hence, we assume λ(M) to be constant in the GHz regime which is of interest here. With this the differential dynamic susceptibility χ can be used to describe the frequency dependency of the delta-E effect. For the specific situation of a hard axis magnetization process the projection $m_{0}\left(H\right)$ can be eliminated from Equation (5) with the Stoner–Wohlfarth model [46]. The solution for a hard axis magnetization process is [44,46]

(6) $$m_{0}\left(H\right)=\left\{\begin{array}{cc} H/H_{K}\; & \left|H\right|<H_{K}\\ 1 & \;\left|H\right|>H_{K} \end{array}\right\}.$$

The effective anisotropy field $H_{K}=2K/\left(\mu_{0}M_{s}\right)$ is expressed via the first order anisotropy constant K. The final solution for the Young’s modulus E(H,f) as a function of the differential susceptibility for this specific case is

(7) $$E\left(H,f\right)=\left\{\begin{array}{cc} 1/E_{m}+1/\Delta E\; & \left|H\right|<H_{K}\\ E_{m} & \;\left|H\right|>H_{K} \end{array}\right\}\;\mathrm{with}\;\frac{1}{\Delta E}=\frac{9}{4}\frac{\mu_{0}\lambda_{s}^{2}H^{2}}{K^{2}}\chi \;.$$

### 2.3. Dynamic Differential Susceptibility

For the differential dynamic susceptibility, a single spin model is used. The motion of the magnetic moments is described by the Landau–Lifshitz–Gilbert equation [47]:

(8) $$\frac{\partial \bar{M}}{\partial t}=\gamma \bar{M}\times {\bar{H}}_{\mathrm{eff}}+\frac{\alpha }{M_{s}}\bar{M}\times \frac{\partial \bar{M}}{\partial t}\;.$$

In the equation, γ is the gyromagnetic ratio, α is the Gilbert damping parameter, ${\bar{H}}_{\mathrm{eff}}$ is the effective field vector and $\bar{M}$ is the magnetization vector. The components of the effective field result from the energy density function u, which is given in the Appendix A. For u we consider an effective uniaxial energy density, a Zeeman term and a demagnetizing energy density. The components $H_{\mathrm{eff},i}$ of the effective field are then given by

(9) $$H_{\mathrm{eff},i}=H_{K}\;\left(m_{0,1}ea_{1}+m_{0,2}ea_{2}+m_{0,3}ea_{3}\right)ea_{i}+H_{i}+H_{d,i}\;\mathrm{with}\;i=1,2,3\;.$$

The components $H_{d,i}=D_{ii}M_{s}m_{0,i}$ of the demagnetizing field are given by the product of the respective component $m_{0,i}$ of the normalized magnetization and the component $D_{ii}$ of the diagonal demagnetizing tensor D. The direction cosines of the easy axis vector are given by $ea_{i}$. $H_{i}$ are the components of the applied static magnetic field vector.

The spin dynamic is linearized using a procedure similar to the one commonly used for ferromagnetic resonance (FMR) calculations [48,49]. In our case, the effective AC driving field is aligned with the magnetic bias field. The fact that the AC effective field originates from an external stress and not from an external magnetic field does not change the calculation procedure. ${\bar{m}}_{0}$ can be obtained from minimizing the energy density u numerically or from Equation (6) in the ideal hard axis case. The following expression is the result for the component $\chi_{11}≔\chi$ of the dynamic differential magnetic susceptibility along the magnetic bias field

(10) $$\chi =\frac{\gamma M_{s}\;m_{0,2}^{2}}{\gamma \left(H_{\mathrm{eff}}+M_{s}\tilde{D}-H_{K}{\tilde{ea}}^{2}-\alpha i\omega /\gamma \right)-\frac{\omega^{2}}{\gamma \left(H_{\mathrm{eff}}+M_{s}D_{33}-\frac{\alpha i\omega }{\gamma }\right)}}\;,$$

with $\tilde{D}≔D_{11}m_{0,2}^{2}+D_{22}m_{0,1}^{2}$, $\tilde{ea}≔ea_{1}m_{0,2}+ea_{2}m_{0,1}$ and the angular frequency ω=2πf of the driving field. The magnitude $H_{\mathrm{eff}}$ of the effective field is given by $H_{\mathrm{eff}}={\left(H_{\mathrm{eff},1}^{2}+H_{\mathrm{eff},2}^{2}+H_{\mathrm{eff},3}^{2}\right)}^{1/2}$ with $H_{\mathrm{eff},i}$ from Equation (9). The full tensor of the differential susceptibility and more details on the calculation procedure are given in the Appendix A. Together with Equation (5) or Equation (7), Equation (10) can be used to describe the frequency and magnetic field dependency of the Young’s modulus. Results are shown and discussed in the following section.

## 3. Results and Discussion

In this section, we first present results of the dynamic susceptibility and the Young’s modulus E(H,f) from an ideal single-spin. Afterwards, a mean-field model is used to compare the modeled magnetization M(H) and the ferromagnetic resonance frequency $f_{\mathrm{FMR}}$ with measurements. The extracted parameters are used to calculate the magnetic part $\partial_{E,H}≔\partial E/\partial H$ of the sensitivity of delta-E effect sensors as a function of damping parameter α and operation frequency f.

For all calculations in this section, we consider a thin film for which the approximation $D_{11}\approx 0\;,\;D_{22}\approx 0$ and $D_{33}\approx 1$ holds well. For the material parameters we consider the gyromagnetic ratio $\gamma =2.21\times 10^{-5}\;\mathrm{Hz}/\left(A/m\right)$ [50] of the free electron, a saturation magnetostriction of $\lambda_{s}=35\;\mathrm{ppm}$ [42] and a saturation Young’s modulus of $E_{m}=150\;\mathrm{GPa}$ [42]. We use $K=1400\;J/m^{3}$ and a saturation flux density of $\mu_{0}M_{s}=1.48\;T$, obtained from the measurements in Section 3.2. 

### 3.1. Frequency Dependency of the Young’s Modulus and Dynamic Susceptibility 

In the following section, we illustrate the frequency and magnetic bias field dependency of the differential dynamic susceptibility χ and the Young’s modulus E. Calculations are made with the example of an ideal hard-axis magnetization process of a single-spin. For the Young’s modulus Equation (7) is used with the dynamic differential susceptibility χ from Equation (10). A damping parameter of α=0.02 is used.

**Figure 2:**
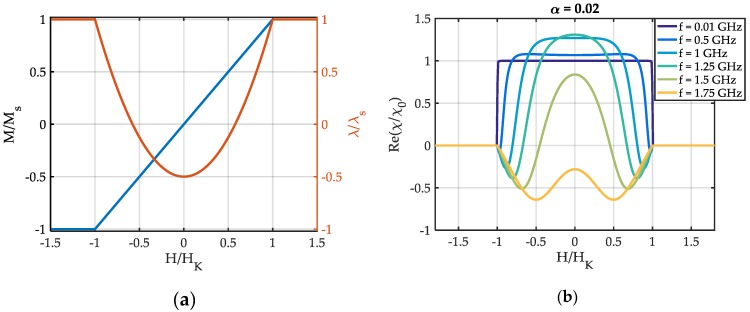
(**a**) Modelled magnetization component M along the magnetically hard axis, normalized to the value $M_{s}$ at magnetic saturation and the magnetostrictive strain λ normalized to its value $\lambda_{s}$ at magnetic saturation. (**b**) Modelled real part Re(χ) of the dynamic differential susceptibility χ (Equation (10)), normalized to the static susceptibility $\chi_{0}\approx 639$ of its quasi-static magnetization value at a magnetic bias field H = 0.

In Figure 2a the results for the normalized quasi-static magnetization $M/M_{s}$ and the normalized magnetostrictive strain $\lambda /\lambda_{s}$ are illustrated. They resemble the well-known Stoner–Wohlfarth behavior of a uniaxial anisotropy material. Because M(H) is a linear function for $-H_{K}<H<H_{K}$, the static differential susceptibility $\chi_{0}$ is constant in this magnetic field regime. The real part Re{χ} of the differential dynamic susceptibility χ (Figure 2b) is consistent with the quasi-static solution at low frequencies. With increasing frequency, the discontinuity around $H=H_{K}$ is rounded off and Re{χ} develops two minima. The minima occur due to the continuous shift of the ferromagnetic resonance frequency from $f_{\mathrm{FMR}}=0\;\mathrm{Hz}$ at $H=\pm H_{K}$ (due to the simple single-spin model) up to about $f_{\mathrm{FMR}}=1.65\;\mathrm{GHz}$ at H = 0. The result at H = 0 is equal to the result from the equation of Kittel [51].

**Figure 3:**
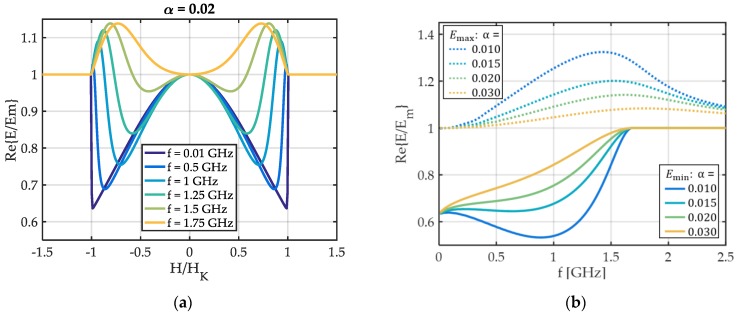
Results for the real part Re(E) of the Young’s modulus E, normalized to its value $E_{m}$ at magnetic saturation. (**a**) As a function of the magnetic bias field for different excitation frequencies f and (**b**) the evolution of maxima and minima visible in (**a**) with the excitation frequency f.

In Figure 3a the real part Re{E} of the Young’s modulus is plotted, normalized to its value $E_{m}$ at magnetic saturation. At low frequencies (f=0.01 GHz), Re{E(H)} is consistent with the results from quasi-static single-spin models [2,28]. With increasing excitation frequency f, the curve rounds out and develops two maxima $E_{\max}$ in addition to the minima $E_{\min}$ present at quasi static *f*. These maxima have been observed experimentally in a previous study [22]. The change of the maxima $E_{\max}$ and minima $E_{\min}$ with frequency depends strongly on the damping factor α as shown in Figure 3b. For α=0.03, $E_{\min}$ continuously increases with f. For smaller α, a minimum occurs in $E_{\min}\left(f\right)$ that is more pronounced for lower damping and shifted to higher f. The maximum of $E_{\max}$ increases for smaller α and shifts to lower frequencies.

**Figure 4:**
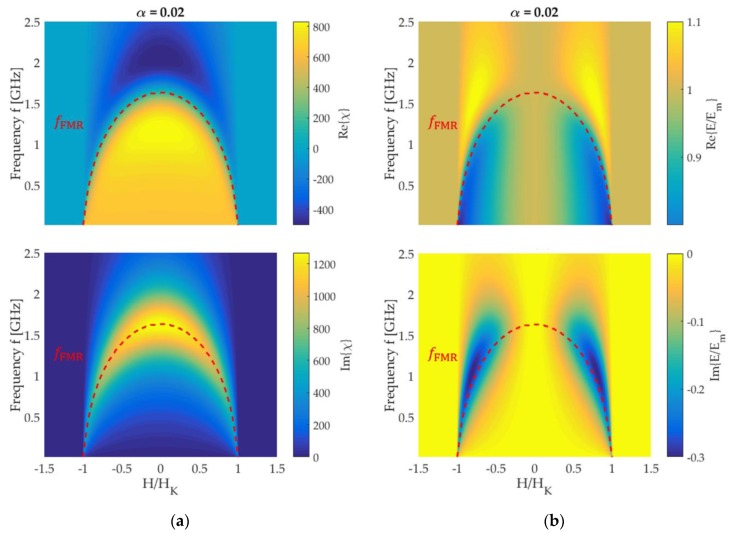
Model results for an ideal hard axis magnetization process: (**a**) real part Re{χ} (top) and imaginary part Im{χ} (bottom) of the differential dynamic susceptibility χ. (**b**) The real part Re{E/Em} (top) and the imaginary part $Im\left\{E/E_{m}\right\}$ of the normalized Young’s modulus $E/E_{m}$ as a function of the external magnetic field and the frequency. A damping factor of α=0.02 is used. The ferromagnetic resonance frequency $f_{\mathrm{FMR}}$ defined by the maximum of Im{χ} is denoted by a red dashed line. Because Im{χ}=0 for $H>\left|H_{K}\right|$, $f_{\mathrm{FMR}}$ is not shown in this field regime.

The complete dependency of the complex E(H,f) and χ(H,f) are shown in Figure 4. The behavior of the Young’s modulus with frequency can be understood by considering the ferromagnetic resonance frequency $f_{\mathrm{FMR}}$. The $f_{\mathrm{FMR}}$ is obtained from the maximum of the imaginary part Im{χ(f)} of the dynamic differential susceptibility χ (Figure 4a, bottom) in frequency f. A dashed red line in Figure 4a(bottom) shows how the $f_{\mathrm{FMR}}$ changes with the magnetic bias field H and f. Starting at H=0, $f_{\mathrm{FMR}}$ continuously decreases until $\left|H\right|=H_{K}$. The decrease occurs, because the AC magnetization always oscillates in a plane perpendicular to ${\bar{m}}_{0}$. Hence, it oscillates along the hard axis initially at H = 0. With rotation of ${\bar{m}}_{0}$, the AC magnetization increasingly oscillates along the easy axis. Consequently, the restoring force reduces and $f_{\mathrm{FMR}}$ decreases. For $\left|H\right|>H_{K}$, Im{χ} and Re{χ} are zero because $m_{0,2}=0$ in Equation (10). At the ferromagnetic resonance frequency $f_{\mathrm{FMR}}$, the sign of the real part Re{χ} of χ changes and becomes negative above it (Figure 4a, top). Consequently, the change of sign occurs also at $f_{\mathrm{FMR}}$ in Re{ΔE} as defined in Equation (4). Though, because of the inversion, the frequency at which $Re\{E/E_{m}\}=1$ is at frequencies $f>f_{\mathrm{FMR}}$ in Figure 4b (top). The same applies for the maximum of $Im\{E/E_{m}\}$ in Figure 4b (bottom). 

### 3.2. Mean-Field Calculations and Measurements

At $\left|H\right|=H_{K}$ the discontinuity in M(H) results in a discontinuity in Re{E} as well. Hence, the sensitivity S∝∂Re{E}/∂H is not defined at $H_{K}$ within the single-spin model. In real magnetic films, distributions of the magnetization may occur due to inhomogeneities in material, structure or geometry and the resulting variations in the demagnetizing field. By the distributions, the discontinuity in M(H) and E(H) vanishes, which makes these functions fully differentiable. For the mean-field model, we use normal distributions $\delta_{\mathrm{EA}}$ and $\delta_{K}$ of the easy axis (EA) angle and the effective anisotropy energy density K in a single-spin ensemble (Appendix A).

**Figure 5:**
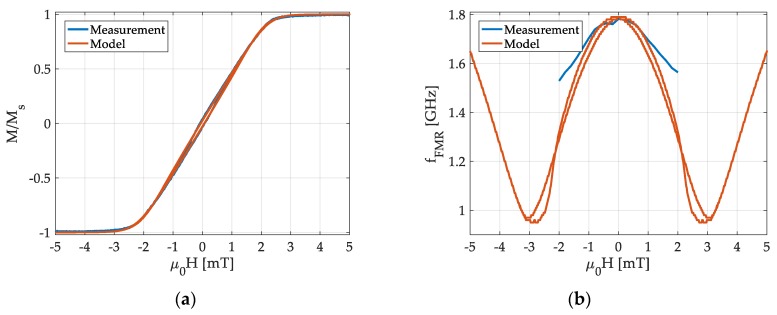
Comparison of measurement and model for (**a**) the normalized magnetization $M\;/M_{s}$ along the magnetically hard axis and (**b**) the ferromagnetic resonance frequency $f_{\mathrm{FMR}}$, defined as the maximum of the imaginary part Im{χ} of the dynamic susceptibility in the model.

To obtain meaningful model parameters, measurements are performed on a 5 × 5 mm sample of 200 nm thick ${\left({\mathrm{Fe}}_{90}{\mathrm{Co}}_{10}\right)}_{78}{\mathrm{Si}}_{12}B_{10}$ with an induced easy axis of anisotropy (Appendix A). The magnetic mean-field model is fitted to quasi-static magnetization measurements performed with a BH-loop tracer. An excellent fit is obtained with $\delta_{\mathrm{EA}}\approx 1\;\%$, $\delta_{K}=15\;\%$ and $K=1400\;J/m^{3}$ (Figure 5a). The other material parameters required are obtained from PIMM measurements. As a result, we obtain a saturation flux density of $\mu_{0}M_{s}=1.48\;T$, a damping constant of α=0.01 and a dynamic anisotropy energy density of $K=1.6\;\mathrm{kJ}/m^{3}$. A discrepancy between the static K and the dynamic K has been observed before [52,53] and was explained with magnetic dispersion [54,55]. This dispersion might also be reflected in the deviation of measured and modelled $f_{\mathrm{FMR}}$ (Figure 5b), which occurs with increasing magnetic field magnitude. The domain structure of the sample is not represented by the single-spin based mean-field model. Additionally, sample misalignment of a few degrees can result in errors. With increasing |H| the component $m_{0,2}$ decreases, until it is too small for a meaningful measurement at large H. For a tilted magnetic easy axis, magnetic saturation along the applied field can never be reached within the Stoner–Wohlfarth model. For such a case it follows that $m_{0,2}>0$, while H→∞. From Equation (10) it is χ→0 because $m_{0,2}\to 0$ with H→∞. Consequently, $f_{\mathrm{FMR}}$ increases due to the stiffening of the magnetic resonator by the increase of the effective field as shown in Figure 5b.

### 3.3. The Magnetic Sensitivity of Delta-E Effect Sensors

**Figure 6:**
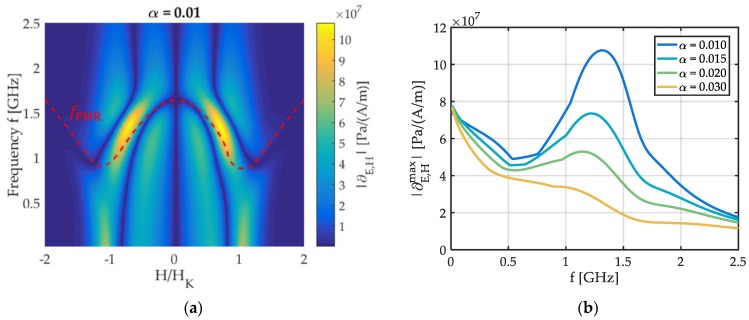
(**a**) Absolute of the derivative $\left|\partial_{E,H}\right|$ of the real part Re{E} of Young’s modulus E with magnetic field H and (**b**) the absolute $\left|\partial_{E,H}^{\max}\right|$ of the maximum of $\left|\partial_{E,H}\left(H\right)\right|$ plotted for various damping parameters α.

Delta-E effect sensors utilize the change of their mechanical resonance frequency $f_{r}$ upon application of a magnetic field for magnetic field sensing. If the Young’s modulus’ contribution to $f_{r}$ dominates, the magnetic part of the sensitivity S results from the derivative $\partial_{E,H}≔\partial E/\partial H$, with $S\propto \partial_{E,H}$. The derivative $\partial_{E,H}$ is calculated using the mean-field model. Due to the distributions introduced, E(H,f) smooths out and the extrema reduce but it does not fundamentally differ from the single-spin result in Figure 4b (top). The absolute $\left|\partial_{E,H}\right|$ of the derivative is shown in Figure 6a for α=0.01, together with $f_{\mathrm{FMR}}$. A global maximum is apparent at frequencies just below $f_{\mathrm{FMR}}\left(H=0\right)$. In Figure 6b the absolute $\left|\partial_{E,H}^{\max}\right|$ of the maximum slope is plotted over the operation frequency f for different damping parameters α. Overall, $\left|\partial_{E,H}^{\max}\right|$ decreases with increasing f, shown, e.g., for α=0.03. For lower α, a local maximum evolves and increases with decreasing damping. At sufficiently low α, the local maximum becomes a global one and exceeds the maximum at quasi-static frequencies.

## 4. Summary and Conclusions

We described the delta-E effect as a function of the dynamic differential susceptibility χ of the magnetization. It can be used to estimate the delta-E effect from static magnetization and magnetostriction measurements if χ is known. A linearization of the Landau–Lifshitz–Gilbert equation was used to describe χ as a function of the magnetization in a single-spin model. χ includes the complete diagonal demagnetizing tensor for a film with uniaxial magnetic anisotropy and an external magnetic field applied in an arbitrary direction. Hence, the model is not restricted to an infinite-plane assumption. The susceptibility calculations match the measurements performed on a typical soft-magnetic FeCoSiB thin film frequently used for magnetic field sensing.

With the extracted material parameters, the Young’s modulus is calculated and discussed as a function of the magnetic field and the operation frequency. A stiffening of the material is observed above the ferromagnetic resonance frequency, which matches with measurements in the literature. The stiffening results from the large phase shift between the oscillating stress and the alternating magnetostrictive strain response. Depending on the damping factors α, the magnitude of the delta-E effect either decreases continuously or exhibits a maximum just below the ferromagnetic resonance frequency.

The model is used to discuss the delta-E effect for magnetic field sensor applications by calculating the magnetic sensitivity of delta-E effect sensors. Like the magnitude of the delta-E effect, a maximum in magnetic sensitivity is visible close to the ferromagnetic resonance frequency for sufficiently small damping factors α. For larger damping factors, the sensitivity continuously decreases with frequency. The results indicate strong influence of the operating frequency on the delta-E effect and the sensitivity, even below the ferromagnetic resonance frequency. Especially for high sensitivity devices, very soft magnetic properties are required that result in a low ferromagnetic resonance frequency. Consequently, the delta-E effect’s frequency dependence should be considered during the design of high sensitivity and high frequency sensors. As the delta-E effect occurs in several components of the mechanical stiffness tensor, we expect a similar dependency on the frequency in those components.

## Figures and Tables

**Figure A1:**
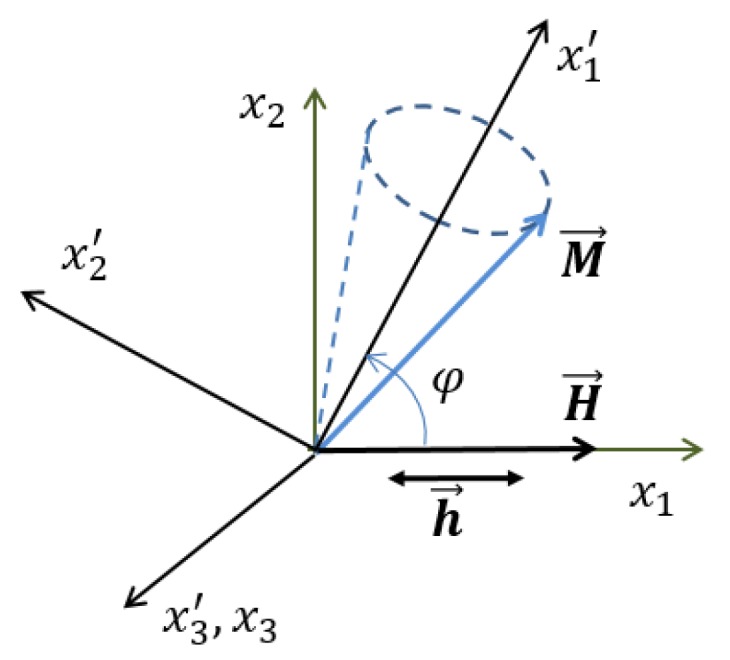
Coordinate system used in the analytical dynamic susceptibility calculation.

## Appendix A

### Appendix A.1. Calculation of the Dynamic Suceptibility

For the calculation of the dynamic susceptibility we used two coordinate systems: a global one ($x_{1},x_{2},x_{3}$) and a rotated one ($x_{1}^{\prime },x_{2}^{\prime },x_{3}^{\prime }$) as it is illustrated in Figure A1. The rotation angle φ is defined as the angle between the static equilibrium vector $\bar{M}$ of magnetization ${\bar{M}}^{*}$ and the $x_{1}$-axis. Along the $x_{1}$-axis an external magnetic field ${\bar{H}}_{\mathrm{app}}^{*}=\bar{H}+\bar{h}$ is applied with a static part $\bar{H}$ and a dynamic part $\bar{h}$.

The effective field ${\bar{H}}_{\mathrm{eff}}^{*}$ and the rotation angle φ are obtained from the energy density u:

(A1) $$u=K-\frac{1}{M_{s}^{2}}K{\left({\bar{M}}^{*}\bar{ea}\right)}^{2}+\frac{1}{2}\mu_{0}{\bar{M}}^{*}D{\bar{M}}^{*}-\mu_{0}{\bar{H}}_{\mathrm{app}}^{*}{\bar{M}}^{*}.\;$$

For the following calculations we separate the magnetization vector ${\bar{M}}^{*}=\bar{M}+\bar{m}$ into a static part $\bar{M}$ and a dynamic part $\bar{m}$. Correspondingly, the effective field vector ${\bar{H}}^{*}={\bar{H}}_{\mathrm{eff}}+{\bar{h}}_{\mathrm{eff}}$ is expressed as a sum of a static effective field ${\bar{H}}_{\mathrm{eff}}$ and a dynamic one ${\bar{h}}_{\mathrm{eff}}$. In equilibrium, $\bar{M}$ lies along the static part ${\bar{H}}_{\mathrm{eff}}$ of the effective field vector. Hence, in the rotated coordinate system ($x^{\prime }$) the only non-zero component of ${\bar{H}}_{\mathrm{eff}}^{\prime }$ is $H_{\mathrm{eff},1}^{\prime }=H_{\mathrm{eff}}$ and of ${\bar{M}}^{\prime }$ is $M_{1}^{\prime }=\;M_{s}$. The dynamic vectors $\bar{m}$ and ${\bar{h}}_{\mathrm{eff}}$ can be described with harmonic oscillations, presuming that the amplitude of the precession is small. In rotated coordinates the complete vectors are

(A2) $${\bar{H}}^{*\prime }=\left(\begin{array}{c} H_{\mathrm{eff}}\\ 0\\ 0 \end{array}\right)+\left(\begin{array}{c} h_{\mathrm{eff},\;1}^{\prime }\\ h_{\mathrm{eff},\;2}^{\prime }\\ h_{\mathrm{eff},\;3}^{\prime } \end{array}\right)e^{i\omega t},\;{\bar{M}}^{*\prime }=\left(\begin{array}{c} M_{s}\\ 0\\ 0 \end{array}\right)+\left(\begin{array}{c} 0\\ m_{2}^{\prime }\\ m_{3}^{\prime } \end{array}\right)e^{i\omega t},$$

with the dynamic components $h_{\mathrm{eff},\;i}^{\prime }e^{i\omega t}$ of the effective field vector and the corresponding dynamic magnetization components , both in rotated coordinates.

Substituting ${\bar{H}}^{*\prime }$ and ${\bar{M}}^{*\prime }$ into Equation (8) the LLG equation is rearranged to obtain the components $\chi_{ij}^{\prime }≔\;m_{i}^{\prime }/h_{j}^{\prime }$ of the dynamic susceptibility tensor $\chi^{\prime }$ in the rotated coordinate system. The harmonic approximation only holds for small oscillations ($m_{i}^{\prime }\ll M_{s},\;h_{\mathrm{eff},i}^{\prime }\ll H_{\mathrm{eff}}$) of the magnetization and the effective field. As a consequence, all terms $m_{i}^{\prime }h_{\mathrm{eff},i}^{\prime }\approx 0$ in the cross product of the LLG equation (Equation (8)), which results in all $\chi_{i1}^{\prime }=0$. Furthermore, the plane of magnetization precession is perpendicular to $\bar{M}$ and the driving field $\bar{h}$ is perpendicular to the $x_{3}^{\prime }$ axis. Hence, all components $\chi_{1j}^{\prime }$ and $\chi_{i3}^{\prime }$ vanish as well. The residual rotated non-zero components are:

(A3) $$\chi_{22}^{\prime }=\frac{\gamma M_{s}/\left[\gamma H_{\mathrm{eff}}+\gamma M_{s}D^{\prime }-\gamma H_{K}ea\prime^{2}-\alpha i\omega \right]}{1-\frac{\omega^{2}}{\left[\gamma H_{\mathrm{eff}}+\gamma M_{s}\tilde{D}-\gamma H_{K}{\tilde{ea}}^{2}-\alpha i\omega \right]\left(\gamma M_{s}D_{33}+\gamma H_{\mathrm{eff}}-\alpha i\omega \right)}},$$

(A4) $$\chi_{32}^{\prime }=\frac{i\omega \;\gamma M_{s}}{\left[\gamma H_{\mathrm{eff}}+\gamma M_{s}\tilde{D}-\gamma H_{K}{\tilde{ea}}^{2}-\alpha i\omega \right]\left(\gamma M_{s}D_{33}+\gamma H_{\mathrm{eff}}-\alpha i\omega \right)-\omega^{2}},$$

with $\tilde{D}≔D_{11}m_{0,2}^{2}+D_{22}m_{0,1}^{2}$ and $\tilde{ea}≔ea_{1}m_{0,2}+ea_{2}m_{0,1}$. By rotating $\chi^{\prime }$ back to the x-coordinate system, we get the final tensor χ of the dynamic magnetic susceptibility:

(A5) $$\chi =\left(\begin{array}{ccc} {\chi^{\prime }}_{22}{\;\sin}^{2}\varphi & {\chi^{\prime }}_{22}\cos\varphi \;\sin\varphi & 0\\ {\chi^{\prime }}_{22}\cos\varphi \;\sin\varphi & \chi \prime_{22}\cos^{2}\varphi & 0\\ \chi \prime_{32}\sin\varphi & \chi \prime_{32}\cos\varphi & 0 \end{array}\right).$$

In this paper, only uniaxial stress is considered. Therefore, only the $\chi_{11}$ component is used for the Young’s modulus calculations.

### Appendix A.2. Magnetic Mean-Field Model

For the simulation, we consider an ensemble of single-domain particles. Each particle has a different orientation of its easy axis vector $\bar{ea}$ to the external magnetic field $\bar{H}$ and an anisotropy energy density K. The orientations of $\bar{ea}$ and values of K are taken to be normally distributed with a standard deviation $\delta_{\mathrm{EA}}$ of the easy axis and a standard deviation $\delta_{K}$ of K. The global magnetization curve is obtained by averaging the magnetization over all particles. For the delta-E effect, the Young’s modulus is calculated for each particle and then averaged over all particles to obtain the global Young’s modulus. The normalized static magnetization vector ${\bar{m}}_{0}$ is obtained for each particle by minimizing its energy density function *u* (Equation (A1)), setting the dynamic components of ${\bar{h}}^{\prime }$ and ${\bar{m}}^{\prime }$ in Equation (A2) to zero.

### Appendix A.3. Model Parameters for Figure 1

For the calculations shown in Figure 1 (Section 2.1) we used a standard deviation $\delta_{K}=30\%$ around a mean of $K=1.9\;\mathrm{kJ}/m^{3}$ and a standard deviation $\delta_{\mathrm{EA}}=1\;\%$ of the easy axis. A size of $\left(3\times 1\times 0.002\right)\;{\mathrm{mm}}^{3}$ is used for the magnetic layer, corresponding to the dimensions given in [7]. These dimensions result in $D_{11}=2.4223\cdot 10^{-4}\;,\;D_{22}=15\cdot 10^{-4}$ and $D_{33}=0.9983$ for the ballistic demagnetizing factors in the center of a rectangular prism [56]. Except of K, we used the same material parameters as given in Section 3.

### Appendix A.4. Measurements

Experiments are performed on a 5 × 5mm sample of 200nm thick ${\left({\mathrm{Fe}}_{90}{\mathrm{Co}}_{10}\right)}_{78}{\mathrm{Si}}_{12}B_{10}$ on a Si substrate. A uniaxial anisotropy is induced by magnetization during the deposition. Quasi static magnetization curves are measured by a BH-loop tracer at 10Hz. FMR frequencies are measured by pulsed inductive microwave magnetometry (PIMM) [57] in flip chip geometry on a coplanar waveguide (CPW). The hard axis of the sample is aligned parallel to the Oersted field of the CPW and magnetic bias fields are also applied parallel to the Oersted field of the CPW. Each bias field is reached starting from positive saturation of the sample and lowering it to the desired field strength to perform the PIMM measurement. The magnetic field strength of the Oersted field is ca. 3A/m. The FMR frequency for each bias field is extracted by the maximum of the magnitude of the Fourier transformed time signal.

## References

[1] Kneller E. (1962). Ferromagnetismus.

[2] Livingston J.D. (1982). Magnetomechanical properties of amorphous metals. Phys. Status Solidi A.

[3] Lee E.W. (1955). Magnetostriction and Magnetomechanical Effects. Reports Prog. Phys..

[4] Gojdka B., Jahns R., Meurisch K., Greve H., Adelung R., Quandt E., Knöchel R., Faupel F. (2011). Fully integrable magnetic field sensor based on delta-E effect. Appl. Phys. Lett..

[5] Jahns R., Zabel S., Marauska S., Gojdka B., Wagner B., Knöchel R., Adelung R., Faupel F. (2014). Microelectromechanical magnetic field sensor based on the delta-E effect. Appl. Phys. Lett..

[6] Zabel S., Kirchhof C., Yarar E., Meyners D., Quandt E., Faupel F. (2015). Phase modulated magnetoelectric delta-E effect sensor for sub-nano tesla magnetic fields. Appl. Phys. Lett..

[7] Zabel S., Reermann J., Fichtner S., Kirchhof C., Quandt E., Wagner B., Schmidt G., Faupel F. (2016). Multimode delta-E effect magnetic field sensors with adapted electrodes. Appl. Phys. Lett..

[8] Nan T., Hui Y., Rinaldi M., Sun N.X. (2013). Self-biased 215 MHz magnetoelectric NEMS resonator for ultra-sensitive DC magnetic field detection. Sci Rep.

[9] Hui Y., Nan T., Sun N.X., Rinaldi M. (2015). High resolution magnetometer based on a high frequency magnetoelectric MEMS-CMOS oscillator. J. Microelectromechanical Syst..

[10] Li M., Matyushov A., Dong C., Chen H., Lin H., Nan T., Qian Z., Rinaldi M., Lin Y., Sun N.X. (2017). Ultra-sensitive NEMS magnetoelectric sensor for picotesla DC magnetic field detection. Appl. Phys. Lett..

[11] Tu C., Chu Z.-Q., Spetzler B., Hayes P., Dong C.-Z., Liang X.-F., Chen H.-H., He Y.-F., Wei Y.-Y., Lisenkov I. (2019). Mechanical-Resonance-Enhanced Thin-Film Magnetoelectric Heterostructures for Magnetometers, Mechanical Antennas, Tunable RF Inductors, and Filters. Materials.

[12] Hanna S.M. (1987). Magnetic Field Sensors Based on SAW Propagation in Magnetic Films. IEEE Trans. Ultrason. Ferroelectr. Freq. Control.

[13] Liur X. (2016). Enhanced sensitivity of temperature- compensated SAW-based current sensor using the magnetostrictive effect. Smart Mater. Struct..

[14] Polewczyk V., Dumesnil K., Lacour D., Moutaouekkil M., Mjahed H., Tiercelin N., Petit Watelot S., Mishra H., Dusch Y., Hage-Ali S. (2017). Unipolar and bipolar high-magnetic-field sensors based on surface acoustic wave resonators. Phys. Rev. Appl..

[15] Kittmann A., Durdaut P., Zabel S., Reermann J., Schmalz J., Spetzler B., Meyners D., Sun N.X., McCord J., Gerken M. (2018). Wide Band Low Noise Love Wave Magnetic Field Sensor System. Sci. Rep..

[16] Liu X., Tong B., Ou-Yang J., Yang X., Chen S., Zhang Y., Zhu B. (2018). Self-biased vector magnetic sensor based on a Love-type surface acoustic wave resonator. Appl. Phys. Lett..

[17] Mazzamurro A., Talbi A., Dusch Y., Elmazria O., Pernod P., Matar O.B., Tiercelin N. (2018). Highly Sensitive Surface Acoustic Wave Magnetic Field Sensor Using Multilayered TbCo2/FeCo Thin Film. Proceedings.

[18] Webb D.C., Forester D.W., Ganguly A.K., Vittoria C. (1979). Applications of amorphous magnetic-layers in surface-acoustic-wave devices. IEEE Trans. Magn..

[19] Robbins W.P., Hietala A. (1988). A Simple Phenomenological Model of Tunable SAW Devices Using Magnetostrictive Thin Films. IEEE Trans. Ultrason. Ferroelectr. Freq. Control.

[20] Wiegert R.F., Levy M. (1989). Enhanced magnetically tunable attenuation and relative velocity of 0.6 GHz Rayleigh waves in nickel thin films. Appl. Phys. Lett..

[21] Wiegert R.F. (2002). Magnetoelastic surface acoustic wave attenuation and anisotropic magnetoresistance in Ni[sub 3]Fe thin films. J. Appl. Phys..

[22] Smole P., Ruile W., Korden C., Ludwig A., Quandt E., Krassnitzer S., Pongratz P. Magnetically tunable SAW-resonator. Proceedings of the IEEE International Frequency Control Symposium and PDA Exhibition Jointly with the 17th European Frequency and Time Forum.

[23] Sun N.X., Srinivasan G. (2012). Voltage Control of Magnetism in Multiferroic Heterostructures and Devices. Spin.

[24] Liu X., Ou-Yang J., Tong B., Chen S., Zhang Y., Zhu B., Yang X. (2019). Influence of the delta-E effect on a surface acoustic wave resonator. Appl. Phys. Lett..

[25] Mason W.P. (1949). Domain Wall Relaxation in Nickel. Phys. Rev..

[26] Johnson S.J., Rogers T.F. (1952). Magnetically induced ultrasonic velocity changes in polycrystalline nickel. J. Appl. Phys..

[27] Mason W.P. (1953). Rotational Relaxation in Nickel at High Frequencies. Rev. Mod. Phys..

[28] Squire P.T. (1990). Phenomenological model for magnetization, magnetostriction and delta-E effect in field-annealed amorphous ribbons. J. Magn. Magn. Mater..

[29] Squire P.T. (1995). Domain model for magnetoelastic behaviour of uniaxial ferromagnets. J. Magn. Magn. Mater..

[30] Datta S., Atulasimha J., Mudivarthi C., Flatau A.B. (2010). Stress and magnetic field-dependent Young’s modulus in single crystal iron-gallium alloys. J. Magn. Magn. Mater..

[31] Bou Matar O., Robillard J.F., Vasseur J.O., Hladky-Hennion A.C., Deymier P.A., Pernod P., Preobrazhensky V. (2012). Band gap tunability of magneto-elastic phononic crystal. J. Appl. Phys..

[32] Zhou H., Talbi A., Tiercelin N., Bou Matar O. (2014). Multilayer magnetostrictive structure based surface acoustic wave devices. Appl. Phys. Lett..

[33] Daniel L., Hubert O. (2009). An analytical model for the Δ E effect in magnetic materials. Eur. Phys. J. Appl. Phys..

[34] Hubert O., Daniel L. (2010). Measurement and Analytical Modeling of the delta-E Effect in a Bulk Iron-Cobalt Alloy. IEEE Trans. Magn..

[35] Zhang D.G., Li M.H., Zhou H.M. (2015). A general one-dimension nonlinear magneto-elastic coupled constitutive model for magnetostrictive. AIP Adv..

[36] Ganguly A.K., Davis K.L., Webb D.C., Vittoria C. (1976). Magnetoelastic surface waves in a magnetic film-piezoelectric substrate configuration. J. Appl. Phys..

[37] Ganguly A.K., Davis K.L., Webb D.C. (1978). Magnetoelastic surface waves on the (110) plane of highly magnetostrictive cubic crystals. J. Appl. Phys..

[38] Walikainen D., Wiegert R.F., Levy M. Magnetic Field Dependency of 600 MHz SAW Velocity Changes For Thin Ni Films. Proceedings of the Ultrasonics Symposium.

[39] Weiler M., Dreher L., Heeg C., Huebl H., Gross R., Brandt M.S., Goennenwein S.T.B. (2011). Elastically driven ferromagnetic resonance in nickel thin films. Phys. Rev. Lett..

[40] Dreher L., Weiler M., Pernpeintner M., Huebl H., Gross R., Brandt M.S., Goennenwein S.T.B. (2012). Surface Acoustic Wave-Driven Ferromagnetic Resonance in Nickel Thin Films: Theory and Experiment. Phys. Rev. B.

[41] Becker R., Döring W. (1939). Ferromagnetismus.

[42] Ludwig A., Quandt E. (2002). Optimization of the delta E effect in thin films and multilayers by magnetic field annealing. IEEE Trans. Magn..

[43] Sárközi Z., Mackay K., Peuzin J.C. (2000). Elastic properties of magnetostrictive thin films using bending and torsion resonances of a bimorph. J. Appl. Phys..

[44] O’handley R.C. (2000). Modern Magnetic Materials Principles and Applications.

[45] Reid A.H., Shen X., Maldonado P., Chase T., Granitzka P.W., Carva K., Li R.K., Li J., Wu L., Vecchione T. (2018). Beyond a phenomenological description of magnetostriction. Nat. Commun..

[46] Stoner E.C., Wohlfarth E.P. (1948). A Mechanism of Magnetic Hysteresis in Heterogeneous Alloys. Philos. Trans. R. Soc. Lond. Ser. A Math. Phys. Sci..

[47] Gilbert T.L. (2004). A phenomenological theory of damping in ferromagnetic materials. IEEE Trans. Magn..

[48] Gurevich A.G., Melkov G.A. (1996). Magnetizaton Oscillation and Waves.

[49] Panina L.V., Mohri K., Uchiyama T., Noda M. (1995). Giant Magneto-Impedance in Co-Rich Amorphous Wires and Films. IEEE Trans. Magn..

[50] Kraus L. (2003). GMI modeling and material optimization. Sens. Actuators A Phys..

[51] Kittel C. (1948). On the theory of ferromagnetic resonance absorption. Phys. Rev..

[52] Gebert A., McCord J., Schmutz C., Quandt E. (2007). Permeability and Magnetic Properties of Ferromagnetic NiFe/FeCoBSi Bilayers for High-Frequency Applications. IEEE Trans. Magn..

[53] Neudert A., McCord J., Schäfer R., Schultz L. (2004). Dynamic anisotropy in amorphous CoZrTa films. J. Appl. Phys..

[54] Hoffmann H. (1968). Theory of magnetization ripple. IEEE Trans. Magn..

[55] Rantschler J.O., Alexander C. (2003). Ripple field effect on high-frequency measurements of FeTiN films. J. Appl. Phys..

[56] Aharoni A. (1998). Demagnetizing factors for rectangular ferromagnetic prisms. J. Appl. Phys..

[57] Silva T.J., Lee C.S., Crawford T.M., Rogers C.T. (1999). Inductive measurement of ultrafast magnetization dynamics in thin-film Permalloy. J. Appl. Phys..

